# Diagnosis in Dental Practice

**Published:** 1902-01-15

**Authors:** J. Taft


### DIAGNOSIS IN DENTAL PRACTICE.

BY J. TAFT, M.D., D.D.S.

Read before the Ohio State Dental Society, December 5, 1901.

As used in medical literature and practice, diagnosis is the art of ascertaining the presence of abnormal, or more properly, diseased conditions, and to determine the variety and characteristics of disease. The word diagnosis is formed of two Greek words *dia* and *gignosko*. Dia meaning between, through or into. Gignosko, to know. The common definition of this word is, the art or process of discriminating between one disease and another, and distinguishing them by their characteristic signs and symptoms, hence a summary of symptoms with the conclusions attended, determines the distinctive nature of the disease present in any organ or organs of the body.

It may be proper here to inquire, what basal knowledge is required to constitute the accomplished diagnostician. In any of the departments of medical science the art of the general practitioner, and specialist as well, has for a starting point the structure, or anatomy of the body, which should embrace a full knowledge of all the parts or organs, their location, size, form and relation to each other. He who would repair a complicated machine must understand its construction, otherwise, injury and probably ruin would result. It is not only the construction of each part that should be understood, but the individual characteristics and susceptabilities should be fully comprehended. It is a fully demonstrated fact that there is a great variation in the fiber and tone of the tissues of the body in different individuals, and even in the same individual in different environments. While in some they are compact and firmly woven and resistant throughout, npon the other hand there are those in whom the tissues are so defective and feeble in their make-up, as to give but little resistance to disease or its causes. These variations and defects are more pronounced in the soft than in the hard tissues, still in the hard is considerable variety in the quality of the structure. In many cases these peculiarities are not the result of disease, but are concurrent with the production and growth of the organism. The influences that bring about these variations are found in heredity, together with other influences that are operative both before and after birth.

It is not, however, within the scope of this paper to go into the reasons for, nor the causes of the variations that are found in the tone, vigor and make-up of the human body, but simply to state the fact for whomsoever it may interest. The characteristics, peculiarities of the tissues of the body must be recognized and well understood, if we are to understand the susceptabilities and changes that are liable to take place. Not only is it important to possess a thorough knowledge of the various parts, but it is equally important to know the uses of these parts or organs, their functions and modes of action, both normal and abnormal. Thus physiology, as well as anatomy, is a necessity for him who would search out and diagnose the various diseases with which we have to deal. The normal as well as the abnormal should be specially comprehended.

Each of the more important organs of the body has a distinct work to perform, and no one of them ever performs vicarious work, except under stress or failure on the part of some other organ. It is desirable that the dentist should understand these functions, especially in their normal activities, and indeed be so familiar as to recognize and discriminate any perverted or abnormal action; especially when actual disease has or is likely to occur. Thus far should he undoubtedly be able to go, not that he should attempt to treat serious cases, but he should certainly know enough of the various affections to determine their probable influence upon the teeth and various tissues of the mouth and its vicinity, with which he is more specially concerned, and that he may know when to refer such cases to the physician, who, it is presumed, is better able than himself, to care for them.

Again, the dentist should be sufficiently informed in pathology to appreciate the influences of the common diseases and affections upon the teeth, and indeed upon all the structures with which he has to deal.

Without such knowledge the dentist is more or less working in the dark. He knows not whether to postpone or proceed. Neither can he know what the future of the service he renders may be, any prognosis he may venture will be mere guess-work.

The greater ranges of knowledge possessed by the dentist, the better basis will he have for the exercise of his professional skill. It is not the intention here to enter upon the subject of general diagnosis: the caption of this paper is, "Diagnosis in Dental Practice." I

would not, however, have it understood that this expression implies that the dentist should confine his examinations to the mouth and its adjacent parts. He should be well equipped in general diagnostic ability, and use it whenever required in the field of his special observation.

I have thought it well to present the above statement as a basis for special diagnosis, and furthermore it must be borne in mind, that the principles in special or local diagnosis are the same in character as those embraced in examination of the body as a whole.

Inflammation in its characteristics and mode of operation is the same in one structure as in another, modified oftentimes, however, by the peculiarities of the structure in which it is present. For example, in inflammation in the tissues in which there is a large amount of adipose material, there will be less pain, soreness, heat and redness and more swelling and breaking down of structure will occur, but inflammation has the same general character. An abscess presents the same general features in the various structures of the body. An abscess in the finger is exceedingly painful because of the peculiarity of the tissue in which it is found, but the general features are the same as abscess elsewhere in the body. The character of existing causes has much to do in modifying diseased conditions. Some act with great energy and persistence. The virus of poisonous reptiles is of this character. In diagnosis of disease, the variations in the susceptibility must be taken into account if the best results are to be realized. No two persons possess these qualities in exactly the same degree ; nor will the same individual have the same susceptibility under the varying influences and environments to which he is subject. The diagnostician must take these things into account if he is to attain a full knowledge of the conditions with which he is dealing.

### THE WORK OF THE SENSES.

*The eye.* In many conditions the eye will promptly bring the investigation to a definite conclusion without the aid of any of the other senses, thus a variation in the size or volume of a part or parts or of the entire organism, a variation either plus or minus direction, hypertrophy or atrophy will be perceived at once by the trained eye. The movements or motions of organs in a diseased state in many instances, at least, clearly point out the extent and character of abnormal or diseased conditions, for instance, certain movements of the head indicate diseased condition of the brain or of the spinal cord, or of the entire nervous system, especially if certain motions of the limbs are made.

The motions of the body all have their signjficance —a quiver of the chest indicating cardiac perturbation; a rapid expansion and contraction of the chest, as a whole, point clearly to affection of the lungs. Any serious disorder of the organs of digestion will often produce more or less pronounced characteristic movements of the body as a whole. The movement of the eyes oftentimes conveys far more to the physician than can be told by words. His eye takes cognizance of far more than can be expressed by language.

The physician discriminates, it may be, by sight more than by any other sense. Indeed, this is the chief means by which he determines the systemic condition. The distinctive texture and color of the skin and hair is only fully perceived by the sense of sight. The impoverished condition of the blood is detected by sight more readily than by any other sense. The pale, flaccid and exhausted countenance conveys to the physician, and even to the novice, a story that can be told in no other way so emphatically as by the sense of sight. The ruddy glow of health and plump form, the elastic movement, and the glistening eye is apprehended only through vision.

### SENSE OF TOUCH.

Without the sense of touch the physician would be unable to ascertain many facts that are important to him and that must influence in his further procedure; for instance, without the sense of touch, the examination of the pulse to determine faulty action of the heart and vascular system would be impossible. The situation, position, form and size of parts, are in some cases only determined by the tactile sense. This is especially true of parts and organs that are out of the range of vision.

### SENSE OF TASTE.

This is, in some sense, analogous to that of touch. Since the organs of taste must be brought into actual contact with anything upon which its special function is to be exercised, this sense is intimately connected with the digestive apparatus. It is placed at the gateway of the digestive function, in order that it may detect and decide upon the character and quality of whatever is to enter this organism. The discrimination of the quality of substance as made by the sense of taste is very different from that made by any other organ of the body.

### THE SENSE OF SMELL.

Dr. Clark, in the *Medical Recorder*, writing upon the character and function of the sense of smell, gives the following:

"Bernard says that, apart from the excretions, an abnormal odor of the skin tends to draw the flies, and that, however little noticeable it may be, it denotes death is near; and Boerhaave held that a cadaveric odor always presages death. Althaus tells us that Skoda was hardly ever led into error by this inclination, and Compton also laid great stress upon this as a clinical symptom.

"In gout the skin secretions take a special odor which Sydenham compared to whey; it is sour, or at least sourish, and there is an excess of ammonia. In rheumatism it is acetoformic, particularly in the regions of engorged articulations (Monin); it is a sour-smelling, acid perspiration.

"In diabetes the smell is sweetish and mawkish, as of hay, according to Latham, 'acetone,' says Picot, and 'midway between aldehyd and acetone being due to a mixture in variable proportions of the two bodies,' according to Bouchardat.

"A musky odor pertains to several maladies, notably peritonitis, jaundice or icterus; and a stale, sour-beer odor to scrofulosis.

"The pyemic has a sweet, nauseating breath, with perhaps the flavor of new-mown hay.

"In milk-fever the smell is distinctly acid; in typhoid, musty, often with the odor of blood; in typhus, ammoniacal and mouse-like, which latter also obtains in favus; in intermittent, the odor is that of fresh-baked brown bread; yellow fever has a cadaveric smell, or like the washings of a dirty gun-barrel.

"In measles it closely resembles fresh-picked feathers; in diphtheria, is sickening and gangrenous—

an odor that is absolutely pathognomonic ; in smallpox, according to severity and stage, it ranges from that of the fallow deer to the dreadful one of the whole menagerie, or it may be that of burning horn or bones.

"Hysteria usually developes an odor of violets or pineapples ; sudamina, that of putrid straw ; scabies, moldy ; anemia and cholera, ammoniacal. * * *

"Otorrhea has a peculiar, clinging, long-lasting odor that once observed will never be forgotten, so, too, is the odor of a henroost that arises from ozonas and bad chronic catarrhs. Gangrene has an old, dead-meat smell, as have some cancers at certain stages. * * *

"At the onset of the plague the odor is sweet, or honey-like according to Doppner."

### HEARING.

This faculty is valuable in diagnosis in many respects. The condition of the heart is in a large degree determined by the sense of hearing. The experienced diagnostician is able to detect the slightest variation from the normal, to the most pronounced forms of abnormality and diseased conditions.

The conditions both normal and abnormal of the lungs are determined by the hearing. Indeed, this is one of the chief means of ascertaining the condition of the respiratory apparatus.

The principles involved in the above statements will apply, in the main, as well to the practice of the specialist as to the general practitioner. Still, somewhat of details in the diagnosis of dental practice will vary from that of the general practitioner and possibly from that of some other specialists. And, furthermore, that part of the organism to which his work is devoted is the most complicated and sensitive of the system. In many respects this field is intimately connected with the more important organs of the body. How often is it that the entire sympathetic system is involved in extreme suffering, the cause of which is found to be a diseased tooth pulp and alveolar abscess or even dental periostitis? These and other local affections with which the dentist has to deal oftentimes involves the whole system in fever conditions and intensely nervous affections.

Omitting from the above, for the most part, the details of dental diagnosis, it is my earnest desire that these shall be brought out and well considered in the discussion which may follow this paper.

DISCUSSION.

Dr. W. H. Whitslar, of Cleveland: With the advance which has been made in dentistry, diagnosis has become much easier, but this should not cause us to underestimate the importance of it, as the power of diagnosis comes only by hard work and study. The treatment of any disease depends always upon the character of the case. and one's knowledge of the organ or tissue affected may be little or great, which will of course influence the diagnosis. To make a correct diagnosis, one should have a thorough knowledge of all the tissues, organs and functions of the human body; and when a careful diagnosis is made, an adequate fee should be asked for services rendered. In few cases will the fee received come near compensating the cost of procuring the knowledge necessary to render a correct diagnosis. By adequate knowledge is meant more than mere learning, it requires the ability to apply knowledge. Dentistry is the science of the treatment of a portion of the human body, but in this treatment a knowledge of physiology, pathology, bacteriology, and histology is required, and it is the study of such subjects which has made the practice of dentistry a science.

The lack of knowledge in reference to health and disease gives rise to many uncertainties in treatments. Many cases would heal spontaneously as it were, providing they had been allowed to do so. Insight as to nature's methods or conditions of tissues is very necessary.

Nearly all the dental diseases are easy to diagnose because of their expressiveness. If the case is obscure, analysis of the saliva and blood tests may be utilized. Odontalgia is probably the most common disease which the dentist has for treatment. We are often asked what is good for the toothache? One readily recognizes that there are many different causes of toothache. Odontagia is relieved by quinine sometimes, but this would more frequently increase the pain, which emphasizes the fact that a little knowledge is bad, we must know all about the case. One should begin to study the patient the moment he comes into the office, observe external appearance, lips, contour of jaw, and also the face. Examine also every organ and tissue in the mouth; the tongue, its movements and coatings of same for internal disturbances; also the mucous membrane for inflammatory disturbances; the gingiva for all peridental affections; lastly, the teeth, note if any are to be supplied and how those that are in the mouth are used. All these things should be considered together in order to make a proper diagnosis of any condition of the mouth.

Dr. H. A. Smith, of Cincinnati: In reference to diagnosis of dental diseases, the essayist refers to the use of our senses as all important. We all realize this, but do we cultivate them sufficiently? A little while ago the editor of one of the leading dental journals discussed the subject, "The Importance of Diagnosis." He thought the young college student did not give enough attention to it. He said, "Whenever a young physician was called to his first few cases he gave more attention to the remedies than to the diagnosis, and that this probably grew out of his failure to study the science of diagnosis; in other words, he gave more attention to what the professor gave as prescriptions and did not take down the discussions on the manner of diagnosis."

The ability or power to diagnose is not inherent, but it comes only after hard work, and one must always be able to distinguish between the normal and perverted physiology of the human body. The late Dr. Mussey had a most wonderful power of diagnosis; it was often stated that he was endowed, but it was finally ascertained that his great power came only through a thorough system and constant study.

Reference was made to the sense of smell. I think we neglect that very important sense, and the time is coming when disease will be very largely distinguished by smell. It is well known that this sense often tells the quality of the saliva. This important fact is many times overlooked by the student, and is only recalled by suggestion when he meets cases in practice.

Pyorrea alveolaris may be easily distinguished by its odor. I think it smells a good deal like a mouse nest. The sense of smell is many times overlooked because of the use of the sight. I think it is very important, and if I were a young practitioner I would smell every patient.

The sense of taste is also very important. Dr. Atkinson once said that benign pus was good to eat, this surely means something.

The sense of sound is also much neglected by us. Dr. Bodecker in his book on pathology distinguished the degree of inflammation in the tooth also in the periosteum and abscesses, by the sounds emitted, this requires a high degree of cultivation, which we don't all possess. All of our senses should be utilized in a systematic examination of cases, and treatment should be secondary to that and based on the results of the diagnosis.

Dr. C. R. Butler, of Cleveland: One might infer from the manner in which the discussion was opened, that in order to be an expert diagnostician one must be a very learned man, and if he renders services they were worthy of good fees. Considering then the large number of schools which to-day include the study of considerable medicine in their curricula, the students whom they graduate ought to be very expert diagnosticians, but I think it is well known that this is not the fact. To think also that this power comes by magic or any peculiar endowments is superstitious and quackery. Acuteness in diagnosis comes largely by experience and the man himself. It behooves us to be very observing, to use all the faculties we have first. Find out the cause of the trouble first and then look for remedies.

In reference to sound, it is a very important thing, if you practice it a little while you will soon ascertain that there is quite a difference between the sound emitted by striking a live tooth and that by striking a pulpless tooth.

In inflammation of the peridental membrane all physiological conditions must first be well understood before you can diagnose the pathological. When I hear a young man say, "What's the use in studying physiology and pathology to be a dentist?" I know immediately if he starts out so, he will never be a successful dentist. Dr. Taft has given not only general statements with regard to diagnosing, but some specific instructions and suggestions which may be applied scientifically in connection with the use of the various senses. These are all given us to be exercised, and by very little use we shall find that they will be of value, and will lead us to treat cases which come into our hands, more intelligently. The dentist should have a dentist's eye just as the microscopist must have the microscopist's eye. But we should never forget that to obtain these desired results it will require persistent and hard study.

Dr. S. D. Ruggles, of Portsmouth: Diagnosis as practiced by the dentist is really a matter of differentiation. We all know the difference between a live and a pulpless tooth; difference in color, difference in sensations of heat, cold, etc. There are certain characteristics of the gums and teeth which immediately causes us to look for pyorrea. There is nothing a dentist should cultivate more than careful observation, and this must be cultivated as it is not inborne in all of us.

Last Sunday I received a telephone message to call at a certain place to see a man who was suffering with an abscess on the upper left side of the mouth, the tooth had been crowned and there had been a large fistula for a long time but it had now closed up and the tooth was slightly elongated. I came to the conclusion there was pus to be liberated. I took him to the office, cut into the abscess when I succeeded in getting pus. I told him the swelling would go down but that he might have recurrence. The next morning I was telephoned by a physician to come to the patient's house prepared to relieve pain and release pus. I did so, and much to my surprise found the opening free and no pus present.

His face was very much enlarged, and there seemed to be considerable infiltration and color of a light reddish pink with well defined fin-shaped margin; the cellular tissues under eyes were much distended and crept across the bridge of the nose. I knew there was something else than abscess there. I suspected erysipelas, I remembered that in erysipelas these fin-shaped projections crept across the bridge of the nose, etc. Proceeding on this suggestion I commenced a series of questions. I found that the fistula had been present for several years but had caused little or no trouble. I came to the conclusion that it was chronic and that no infection got in through it. After quite a few questions and answers he finally said that the week before he had been to see his father-in-law who had the erysipelas, and I came to the conclusion that his infection came through that source. I told him, therefore, that no pus was there and put in a dressing to take away bad taste, and left him. Just before I left home the doctor diagnosed the case as erysipelas.

Dr. J. Taft: During the discussions the impression has been made upon my mind of the great value of studying the subjects presented to us in papers and discussions. Many times when the subject has been discussed we go home, and except in a general way, forget all about it. All such vital questions as these are worthy of special and continued study. Those who prepare papers for the society, realize how much benefit they get from the study and preparation, as many references must be looked up in text books and dental magazines, which necessarily requires much application.

Observation is employed through the five senses and we often take it for granted that these are in just as good condition as when we received them from our parents. Too much can not be said upon the cultivation of the senses. Suppose the eye had not been trained in order to make a thorough and well-equipped microscopist? Where would microscopy be to-day? This is illustrated in all microscopic work. How much better one who is so trained than one who is not, the latter being unable many times to locate objects under the microscope which would be comparatively easy for the former. This simply emphasizes the importance of the proper cultivation and use of the senses, being careful always to keep them in the best possible condition. How often do we see the dentist going to the operating chair after being in the laboratory, even without cleaning his hands, and if the hands are clean they are very rough. One can not successfully practice both the prosthetic and operative branches of dentistry. We do not realize the value of well-kept hands in operative work. This is manifested by the great lack of care shown by dentists. The sense of smell is also quite important. I remember an old physician who had never used a stethoscope, hearing of it, he finally purchased one. Not understanding its use he began a series of experiments. He was finally called to visit a patient upon whom he thought he could use the stethoscope, and after several trials he placed the large end of it upon the patient's mouth and his nose at the other end. Of course, this was an unreasonable thing to do. but it at least showed a desire on the part of the physician to do something for the patient. If we are all as persevering as this old doctor, in our work, we will learn something we did not know before. The ear should also be cultivated to detect the various sounds. There is a great diversity in the acuteness of physicians to the various sounds of the body; different physicians hearing differently in the same diseases. Some physicians can not tell one tone from another.

In conclusion I trust we shall have received such inspiration from this discussion that we shall go home determined to cultivate our senses and retain them in their best possible condition, so that we may be in condition to look into all our cases of the future with more intelligence than heretofore.

